# Intraoral scanning to fabricate complete dentures with functional borders: a proof-of-concept case report

**DOI:** 10.1186/s12903-019-0733-5

**Published:** 2019-03-13

**Authors:** Alexey Unkovskiy, Eugen Wahl, Anne Teresa Zander, Fabian Huettig, Sebastian Spintzyk

**Affiliations:** 1 Department of Prosthodontics at the Centre of Dentistry, Oral Medicine, and Maxillofacial Surgery with Dental School, Tuebingen University Hospital, Osianderstr. 2-8, 72076 Tuebingen, Germany; 2 Department of Dental Surgery, Sechenov First Moscow State Medical University, Bolshaya Pirogovskaya Street, 19с1, 119146 Moscow, Russia; 3 Section Medical Materials Science and Technology, Tuebingen University Hospital, Osianderstr. 2-8, 72076 Tuebingen, Germany

**Keywords:** Case report, Complete denture; intraoral scanning, Additive manufacturing, 3D printing, Computer aided design

## Abstract

**Background:**

The utilization of intraoral scanning for manufacturing of complete dentures (CD) has been reported recently. However, functional border molding still cannot be supported digitally. A proof-of-concept trial shows two possible pathways to overcome this limitation by integrating a relining procedure into the digital workflow for CD manufacturing.

**Case presentation:**

Intraoral scans and additional facial scans were performed with two various scanning systems for the rehabilitation of an edentulous male patient. The obtained raw data was aligned and used for the computer aided design (CAD) of the CD. The virtually constructed dentures were materialized in two various ways, considering rapid manufacturing and digital relining approaches in order to apply functionally molded borders.

**Conclusion:**

The use of intraoral edentulous jaws scans in combination with the digital relining procedure may allow for fabrication of CD with functional borders within a fully digital workflow.

## Background

During the past decade, prosthetic dentistry was seriously impacted by computer driven technologies, which has also touched upon the rehabilitation of edentulous patients [1]. Fabrication of complete dentures (CD) by means of computer aided design (CAD) and manufacturing (CAM) has been proven to be feasible [2, 3]. A better fit to the underlying tissues [4] and a reduced number of appointments till a final denture delivery [5, 6] were reported to be the main advantages towards the conventional production chain. However, the long-term clinical performance of digitally produced CD was not evaluated until now, and the existing software solutions still require further improvements [7].

Within numerous digital protocols, recently introduced for CD fabrication, CAD/CAM substitutes many of the steps of a conventional production chain [8]. For instance, occlusal rims (OR) and functional impressions (with border moldings) can be digitalized with the use of laboratory scanners for the data input [7, 9]. Then, a denture is constructed virtually in specific CAD software [10, 11]. The facial scans can be obtained for the virtual esthetic evaluation and digital try-in session [9, 12]. A denture prototype can be fabricated by means of additive manufacturing (AM) for the chairside try-in session [8, 13]. A final denture assembly is manufactured using subtractive or additive manufacturing methods [14, 15]. These approaches, however, should be perhaps regarded as “partially digital”, as they consider no intraoral scanning of maxilla and mandible alveolar parts and necessitate analog elements, such as impressions, within the workflow.

In the last years, the intraoral scanning has been widely applied in prosthetic dentistry as an alternative to the conventional impression taking [16–18]. Very recently, a few techniques for the direct capturing of edentulous jaws have been introduced, but do not cover the functional mucosa reflections [19–21]. Furthermore, the reliability and reproducibility of some of these techniques are questionable [22]. The unfeasibility of digital functional impression taking and modest precision are considered herein as main limitations [8, 23]. Some clinical cases have shown the pathway till delivery of the definitive CD: Utilizing intraoral scans in a fully digital workflow either considering no functional borders molding [15], or a using finger to stretch the mucosa and capture its reflections [24]. Such technique was reported to provide a denture with sufficient retention, however, major concern here may be the overextension of the plica intermedia and neglecting of the functional movements.

The present clinical case describes two technical proof-of-concept approaches for fabrication of CDs with functional borders in a fully digital workflow utilizing intraoral scanning.

## Case presentation

**Fig. 1:**
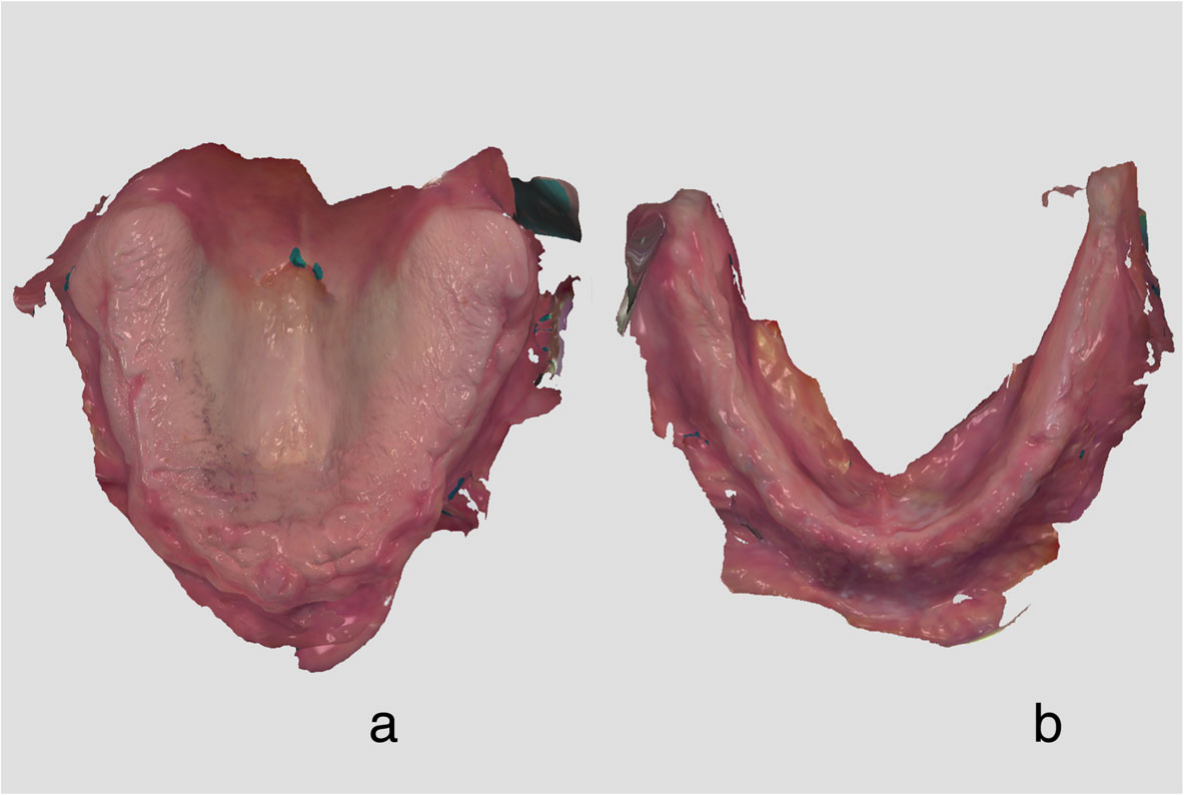
Intraoral scans of edentulous upper (**a**) and lower (**b**) jaws in native format in 3Shape scanning software obtained using the “zig-zag” technique

**Fig. 2:**
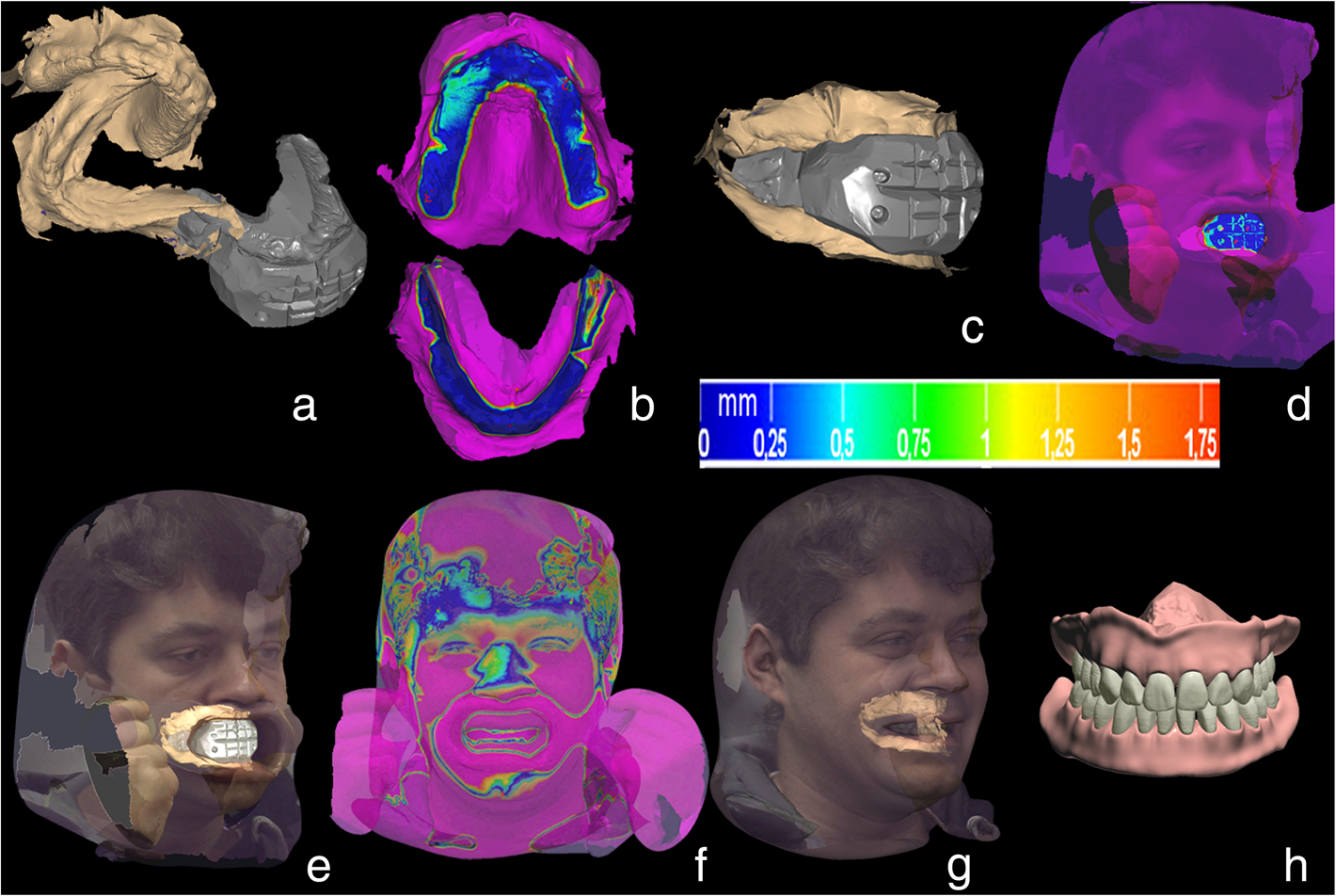
CAD pathway in DentalCAD software (Exocad). **a**: unmatched upper and lower jaws scans and scan of OR; **b**: initial alignment of the both jaw scans with OR using “three points alignment” protocol and the final alignment with “best-fit protocol”. The deviation of the reference areas ranged between 0 and 0.25 mm, as indicated in the scale; **c**: aligned scans of upper and lower jaws with OR; **d**: alignment of OR with the “cheek retractor facial scan” using reference lines on the OR; **e**: aligned OR with the “cheek retractor facial scan” **f**: alignment of the “cheek retractor facial scan” with “smile facial scan” using common reference points (chin, eyes, dorsum nasi); **g**: aligned upper and lower jaw scans embedded into the “smile facial scan” as a result of “**d**” and “**f**”; **h**: the whole dataset was transferred into the Dental System software (3Shape, Copenhagen, Denmark) for the digital design of two dentures

A fully edentulous male patient received conventional CDs and gave his informed written consent to participate in this feasibility trial. The edentulous upper and lower jaws were scanned with the TRIOS3 intraoral scanner (3Shape, Copenhagen, Denmark) (Software version 1.4.7.5) (Fig. 1). The lips were retracted with a Brånemark cheekholder and saliva was constantly removed with an aspirator during intraoral scanning [25]. The “zig-zag” scanning technique was used in this clinical case [24, 26]. The occlusal vertical dimension (OVD) was oriented on the physiological rest position and an occlusion rim (OR) was made with Silaplast putty silicone material (Detax, Ettlingen, Germany). The central, canine, and smile lines were marked with cuts in the silicone on the facial surface of the OR. This OR was digitized extraorally with the same scanner (TRIOS3). In order to capture the facial anatomy, three scans with the OR being placed in the mouth were performed with neutral face, smiling face, and with cheekholders using the priti®mirror scanner (priti®denta, Leinfelden-Echterdingen, Germany). The pathway for raw data matching and virtual denture design using both available dental CAD solutions: DentalCAD software (Version 2.2 Valetta, Exocad, Darmstadt, Germany) and 3Shape Dental System (3shape, Copenhagen, Denmark) is described in Fig. 2. The obtained virtual denture design was used to produce two final denture sets utilizing the following technical approaches.

### “The rapid manufacturing (RM-) approach”

**Fig. 3:**
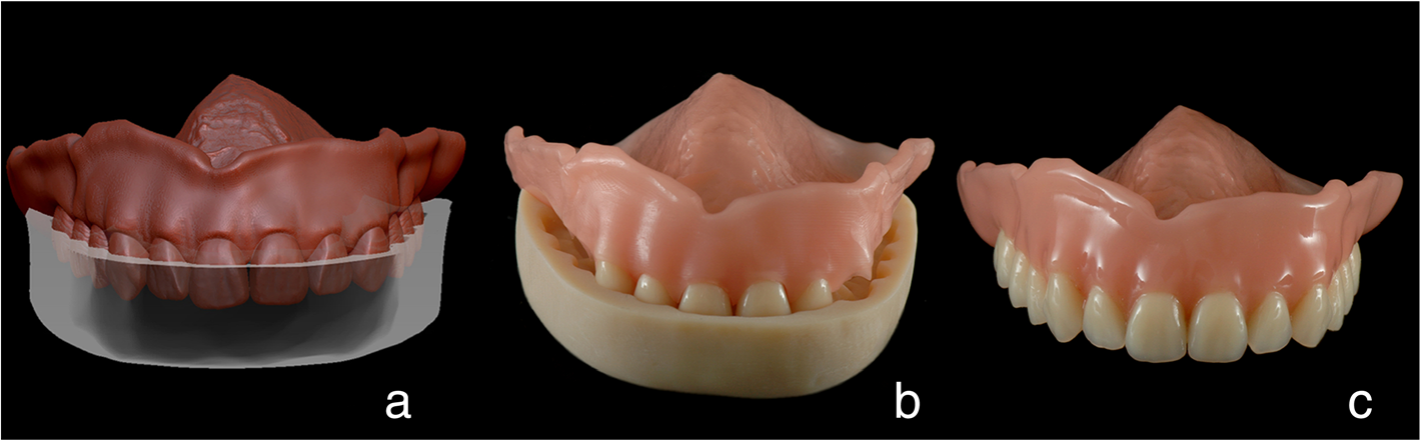
“The rapid manufacturing (RM) approach” (exemplarily upper denture). **a**: the virtual design of a transfer key in Zbrush software (Pixologic, Los Angeles, CA, USA) to ensure the proper teeth positioning; **b**: transfer key put on the denture base and being supported upon the tuber and palatal surfaces; artificial teeth are put into corresponding sockets; **c**: polished denture with artificial teeth in situ being fixed with cold curing PMMA

The virtual models of denture bases without tooth anatomy were milled from Organic PMMA Eco Pink discs (Organical CAD CAM, Berlin, Germany) with Organic Desktop 8 milling unit (Organical CAD CAM, Berlin, Germany). To ensure the proper positioning of the standard artificial teeth (Bonartic NFC+, Candulor, Glattpark, Switzerland) in the denture basis, a transfer key was designed in the Zbrush software (Pixologic, Los Angeles, CA, USA). Figure 3a based on the 3D datasets of the artificial teeth set.

In addition, undercuts on the prostheses’ models were blocked out with a virtual clay tool. An U-shaped bulk was superimposed onto the denture virtual model, so that it covered the artificial teeth, and both retromolar and palatal surfaces. The denture model was cut out from the bulk with the Boolean function constituting the transfer key. This dataset was printed with a direct light processing (DLP) Solflex 170 3D printer (W2P Engineering, Vienna, Austria) using V-Print model material (Voco, Cuxhaven, Germany). Afterwards, the chosen artificial teeth were fixed into the corresponding sockets with the cold curing Aesthetic Blue PMMA (Candulor, Glattpark, Switzerland). The printed transfer key was placed upon the teeth and was pressed onto the denture bases (Fig. 3b). After the PMMA excesses were removed the, dentures were put under pressure (2.5 Bar, 40 °C, 20 min). The dentures were then polished (Fig. 3c).

### “The digital relining (DR) approach”

**Fig. 4:**
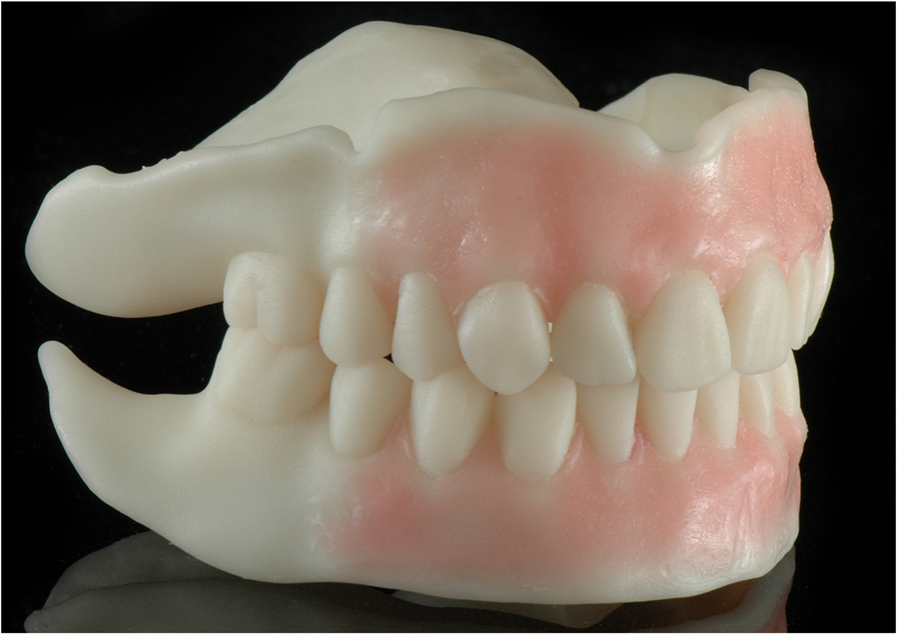
Printed denture prototype with tooth anatomy for the DR approach

This approach encompasses a chairside try-in session. For this purpose, the denture prototype with tooth anatomy was printed with the DLP Solflex 170 3D printer (W2P Engineering, Vienna, Austria) using Solflex prov A2 material (W2P Engineering, Vienna, Austria) (50 μm layer thickness, printing time 5 h). Pink wax (Modelierwachs, Omnident, Rodgau Nieder-Roden, Germany) was applied to imitate the gingiva for a more realistic perception (Fig. 4).

**Fig. 5:**
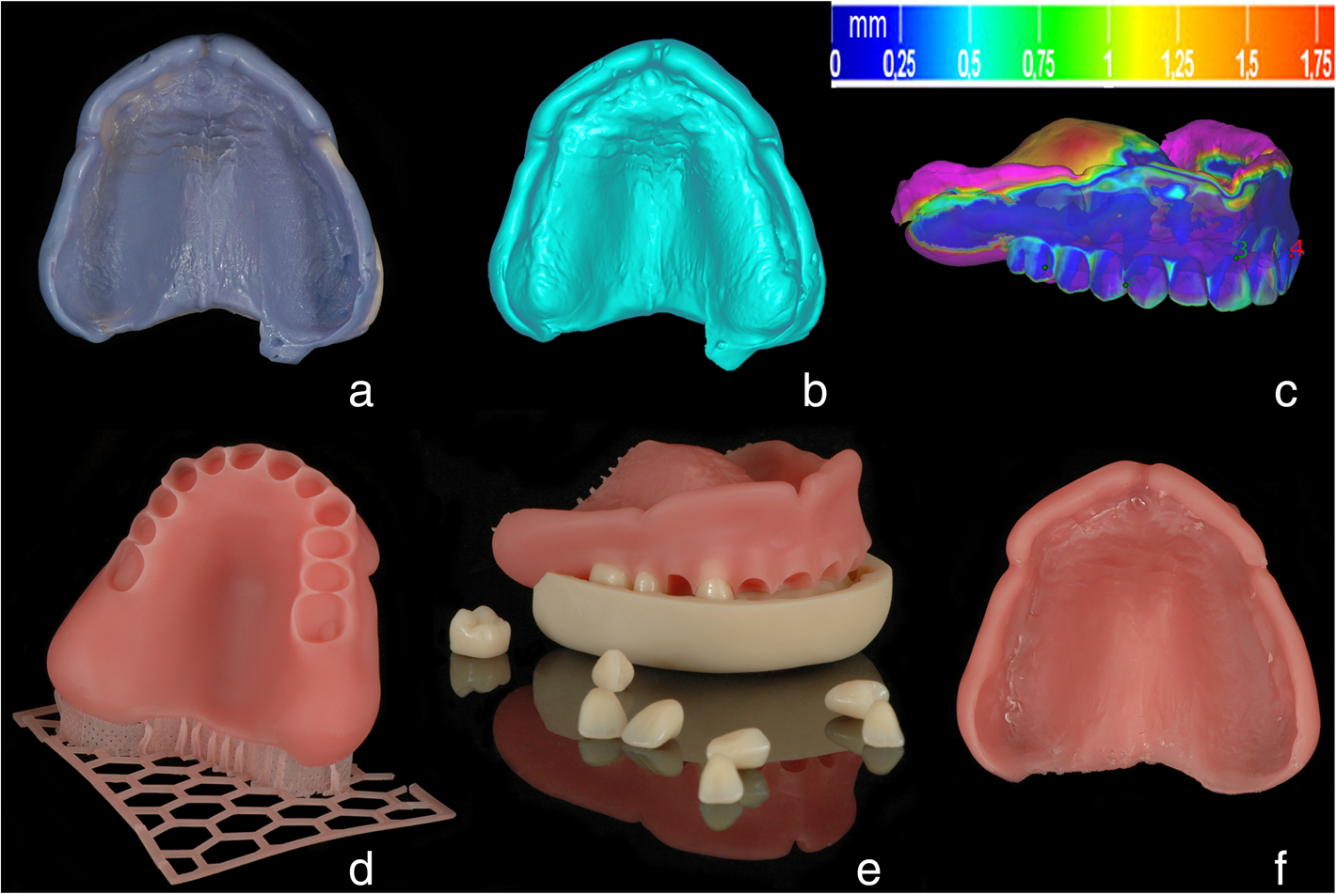
“The digital relining (DR-) approach”. **a**: relined denture prototype; **b**: virtual model of the relined surface obtained with a Trios scanner; **c**: alignment of the relined surface with the existing denture design through the reference teeth and basis surfaces using the best-fit protocol (DentalCAD, Exocad, Darmstadt, Germany); **d**: “digitally relined” denture basis fabricated with Solflex 170 3D printer ‘as is’ (W2P Egineering, Vienna, Austria); **e**: fitting the artificial teeth into the corresponding sockets using a transfer key; **f**: 3D printed final denture surface with functional borders

During the try-in session, the general esthetics and function were checked, including the OVD, occlusion, and the bases extension. Due to suboptimal retention of the prototypes’ bases, they were relined with a Silasoft N silicone material (Detax, Ettlingen, Germany) (Fig. 5a). The silicone excesses that spread over the flanges onto the prototype basis were cut out with a scalpel. The relined prototypes were scanned extraorally with the TRIOS3 scanner (3Shape, Copenhagen, Denmark) (Fig. 5b). The obtained datasets were aligned with the existing 3D denture constructions through the matching surfaces, e.g. teeth and basis in the DentalCAD software (Exocad, Darmstadt, Germany) (Fig. 5c). The matched surfaces were merged together and further adjusted in the Zbrush software (Pixologic, Los Angeles, CA, USA). The relined and adjusted virtual denture models were printed with a Solflex 170 DLP printer (W2P Engineering, Vienna, Austria) with pink resin (Base pink, NextDent, Soesterberg, Netherlands) (50 μm layer thickness, 7 h 50 min printing time for both dentures) (Fig. 5d). The artificial teeth were then fixed into the bases the same way it was done in “RM-approach” described above (Fig. 5e).

## Discussion and conclusions

The digitalization of the CD manufacturing began with the advent of specific CAD software, which allowed for CAM of denture bases and artificial teeth in either a subtractive or additive way [8]. The implementation of the facial scanning into the workflow allowed for the virtual try-in session and contributed to the predictability of the final outcome [11, 12]. The primary data acquisition step, however, remained rather analog and implied conventional impression taking, which was afterwards scanned extraoraly [9]. In 2013, the feasibility of a direct digital impression of edentulous jaws was shown extraoraly [23], however differences up to 591.8 μm to the original model were revealed from this work. With the advent of more sophisticated intraoral scanning devices, the precision of direct digital data acquisition has increased, and minor deviations up to 125 μm were reported [27]. In the last few years, the buccal-occlusal-palatinal (BOP) [26] and “zig-zag” [20] techniques have been mainly used for the intraoral scanning of edentulous jaws. Thereby, the BOP has shown greater trueness but lower precision [28]. A specific pathway was described for an intraoral scanning of a maxillary edentulous jaw considering special markers on the palatal surface to avoid “overlapping effects” [21]. In the present clinical case the intraoral digital impressions of edentulous maxilla were performed with “zig-zag” technique. No visual overlapping effect was detected in the data sets. The scanning process of the edentulous mandible took more time, as it is devoid of anatomical landmarks. Care was taken to retract the cheeks and tongue, to remove the saliva from the mouth floor and keep the palatal surface dry.

Even if the direct scan of the jaws still supports no functional impression, a CD manufactured in such a digital workflow was reported to have a good retention [15]. Thereby, the surface tension between the denture basis and underlying tissue may play a bigger role, than the sealing effect by means of custom formed denture borders [17]. Goodacre et al. have reported that the stretching of the mucosa with the finger, while performing the intraoral scan, may be useful to capture sufficient amount of mucosa reflections and achieve a good peripheral seal. Here, two aspects may be of major concern. First, care must be taken not to overextend the plica intermedia, while manipulating it freely in order to avoid the excessive displacement of the tissue of the vestibule [29]. Second, a traditional functional impression encompasses not only the manual manipulation of the mucosa by the operator, but also the border molding movements performed by the patient himself [30].

Aside from retention, valid orientation of the obtained jaw scans prerequisites a successful, reproducible digital workflow for CD manufacturing. This order implies a transfer of the jaw relation, which is challengeable to perform intraorally. As demonstrated by Kanazawa et al., the reduction of OR dimensions can simplify the scanning process at least for the manufacturing of custom impression trays [31]. However, such an approach hinders the transfer of esthetical lines as well as the alignment and connection of facial and jaw scans. Therefore in the present feasibility study, the OR with the marked esthetical lines was digitalized extraorally and acted as an index object for orientation and CAD alignments. The extraoral scanning process with the intraoral scanner surprisingly was not associated with any difficulties. The OR could be captured with one scanning sequence. For the alignment of OR with the “cheekholder facial scan”, the three-point protocol was used for the initial alignment, followed by the best-fit protocol for the definitive one. The deviation range of the reference areas was between 0 and 0.25 mm, which was considered to be accurate enough to start the CAD stage. During the try-in stage, no deviations in either the occlusion between the prototypes or the orientation of the occlusal plane were revealed. If there should be any inaccuracies in the virtual alignment of the whole dataset, they could be fixed on the try-in stage utilizing the DR approach. However, the RM approach would be more susceptible to any kind of inaccuracies in the data alignment. Minor corrections in terms of OVD and occlusion scheme may be applied chairside. In case of greater deviations, the production process should be started from the very beginning.

**Fig. 6:**
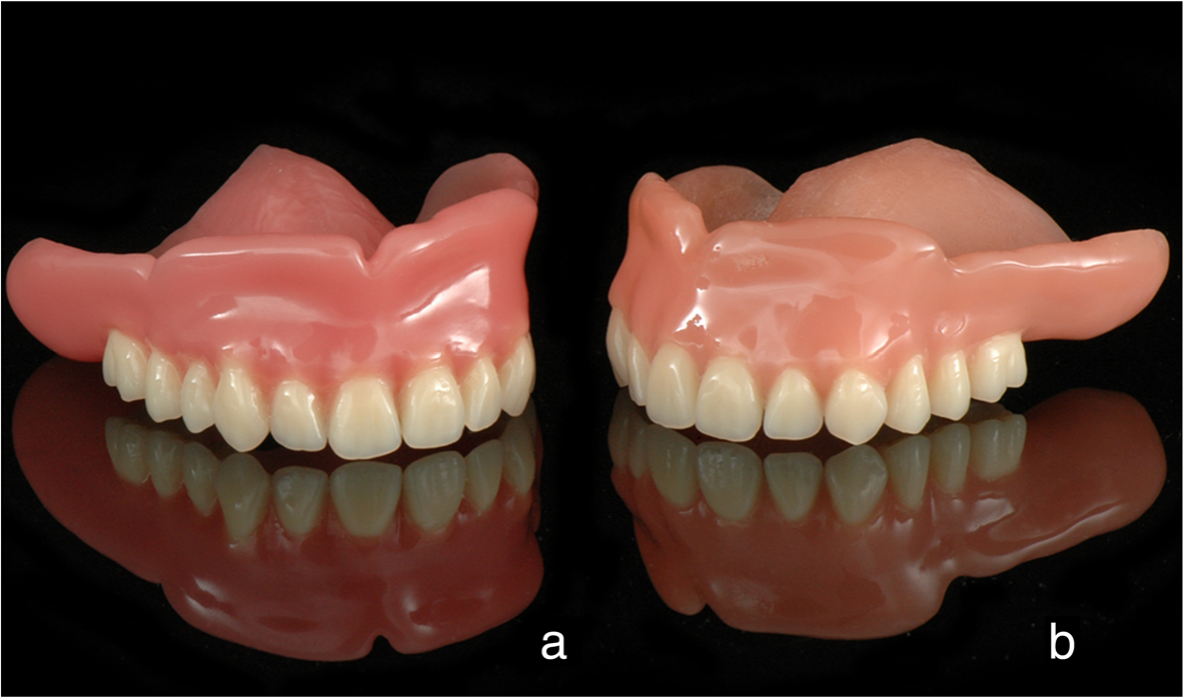
Two upper jaw dentures with custom borders made in a digital workflow utilizing the intraoral jaw scans. **a**: digitally relined and printed (DR approach); **b**: milled and relined in the analog way (RM approach)

The present clinical case shows two pathways to fabricate CD with functional borders in a digital workflow, using intraoral scanning for initial data acquisition and facial scans for the aesthetical adjustment. These techniques result in comparable dentures and require one intraoral scanner, three CAD solutions, and a DLP 3D printer (Fig. 6). Unfortunately, no universal software solution for CAD of CD is available today. Whereas the 3Shape software includes the data library with prefabricated teeth in 3D format, it supports no raw data alignment and integration of the facial 3D scans for the fully 3D smile design. Thus, in order to design a CD with prefabricated teeth (i.e. Candulor), utilizing at the same time the facial 3D scans for the aesthetical adjustment, one is obliged to use a combination of 3Shape and Exocad software solutions.

The RM approach showed the shortest pathway from data acquisition to the final denture delivery within a fully digital workflow in only two appointments. However, as this approach implies no try-in session, the final esthetical outcome is hardly predictable and relies only upon the consistency of the digital try-in session in the CAD software. In the present case, the upper denture fabricated within the RM approach with no border molding showed a poor retention. Thus, a relining procedure with all functional probes can be performed in analog workflow either directly or indirectly [32].

The DR approach considered the virtual relining procedure as an inherent step. This step does not require any additional appointment as it can be performed during the try-in session. The virtual image of the relined surface can be obtained chairside in the same appointment with the intraoral scanner and requires no further hardware investments, i.e. a labor-scanner. A faithful alignment of the scanned relined surface and the existing denture design is of major concern and depend heavily of the square of the matching surface. For this reason, all the silicone excesses that spread over the prototype flanges were cut out with the scalpel in order to increase the matching surface.

The other setback of this approach is the fact that no CAD software today supports the merging procedure of the relined surface with the initial denture design and allows for designing of a transfer index. For this reason, additional software must be used, i.e. the Zbrush, which is capable of aligning and merging of two virtual meshes. Thus, the certain CAD skills and expertise is required. This fact can be considered as the limitation of the described technique and calls for the CAD software enhancement.

The upper denture fabricated within the DR approach demonstrated – as expected – clinically sufficient retention, as the peripheral sealing effect was achieved due to denture flanges customized extension. A perfect retention of the lower denture is commonly hard to achieve [33]. A lingual occlusal scheme without the canine guidance was considered in this case, alternatively to the bilateral balanced occlusion [34, 35].

Within the current technique, the acquired digital set up must be arranged in the CAD software, prior to the prostheses designing process. The setup includes OR, upper and lower jaw scans, a “cheekholder facial scan”, and a “smile scan”. Meaning, the four subsequent alignment procedures may constitute a potential error source. In the present clinical case, the deviation between the matching areas ranged between 0 and 0.25 mm. A systematic study with a greater sample size must address the reliability of this technique.

This clinical trial reveals the current state of technology in a digital workflow of CD fabrication and highlights the possibilities and limitations of the intraoral scans utilization. The RM approach allowed for a quicker workflow within two appointments, but it is questionable as a definitive treatment option due to shortcomings in retention and poor predictability of the esthetic outcome. Thus, the integration of a relining procedure with functional impression – as shown in DR approach – may enhance the denture retention made in a digital workflow, starting with the intraoral scanning. The digital underlining technique shown still implies the conventional functional probes, restricting the unambiguously fully digital workflow.

The two introduced technical approaches revealed that there is still no optimal CAD solution, which would cover such aspects of CD design, as integration of facial scans, fabrication of transfer index, merging of the separate meshes and integration of prefabricated tooth libraries. Furthermore, these approaches require a certain financial investments in both hard- (one intraoral scanner, one facial scanner, one 3D printer) and software (three commercial CAD solutions).

## Abbreviations

CAD: Computer aided design
CAM: Computer aided manufacturing
CD: Complete denture
DR: Digital relining
OR: Occlusal rim
OVD: Occlusal vertical dimension
PMMA: Poly-methylmethacrylate
RM: Rapid manufacturing
